# Co-evaluating health messages through inclusive participatory judging and validation: A public involvement study in Tanzania’s eye health education programme

**DOI:** 10.1186/s12961-026-01519-9

**Published:** 2026-08-07

**Authors:** Asia Haraburda, Frank Sandi, Careen Erick, Christine Sifael, Eden Mashayo, Ving Fai Chan

**Affiliations:** 1 Queen’s University Belfast, University Rd, BT7 1NN, Belfast, Northern Ireland, UK; 2 University of Dodoma Medical School, Dodoma, Tanzania; 3 Vision Care Foundation, Dar-es-Salaam, Tanzania

**Keywords:** Public involvement, Co-production, Participatory action research, Health communication, Eye health, Low- and middle-income countries, Arts-based methods, Disability inclusion

## Abstract

**Background:**

Public involvement in health communication design is becoming increasingly important for creating messages that are inclusive, trusted, culturally resonant and equitable; however, little is known about how communities, including persons with disabilities can take active roles in judging and validating creative health education materials in low-resource settings.

**Objective:**

To explore how inclusive participatory judging and validation can support meaningful community involvement in developing arts-based eye health education materials in Tanzania and to examine participants’ experiences of contributing to these processes.

**Methods:**

A participatory action research (PAR) study using mixed methods to evaluate a nationwide competition that invited local artists to produce short animations and voice dramas to tell key eye health messages. A total of 14 community judges and 17 validation workshop participants assessed submissions using 10 criteria and later validated the top three entries for message inclusion, comprehension and effectiveness.

**Results:**

The involvement of various local stakeholders enabled comprehensive assessment of the effectiveness of the artworks. Recommendations included improving accessibility using subtitles, sign language and clearer visuals. Participants suggested artwork should better reflect the realities of Tanzanian life to enhance their cultural relevance. The participatory judging and validation processes fostered a sense of inclusivity and ownership of the intervention in the community, with judges reporting feeling confident, valued and comfortable.

**Conclusions:**

Participatory judging and validation offered an inclusive and empowering approach to public involvement in health communication design. Beyond improving message quality, these processes fostered dialogue, shared decision-making and community ownership – demonstrating a transferable model for co-producing culturally grounded health education in low- and middle-income settings.

**Supplementary Information:**

The online version contains supplementary material available at https://doi.org/10.1186/s12961-026-01519-9.

## Introduction

Vision impairment remains a major, yet often under-prioritized, public health challenge in Tanzania. Many causes are preventable or treatable, but care-seeking remains low due to social and cultural barriers such as stigma, limited awareness and poor access to services [1–5]. Tanzania does not currently have an effective nationwide eye health promotion strategy and is in need of inclusive educational materials to improve eye service uptake. In this culturally diverse and resource-limited context [6], conventional top-down health promotion may fail to resonate with communities whose beliefs, literacy levels and lived realities shape their understanding of illness and care [4]. Hence, health promotion interventions that are culturally relevant, inclusive, accessible and participatory are essential to bridging the eye health service gap. This has driven growing interest in participatory and arts-based communication strategies that position communities not merely as recipients of information but as active co-producers and co-evaluators of knowledge and solutions [7, 8]. Previously, Participatory Action Research (PAR) has been used to educate Tanzanian youth about human immunodeficiency virus (HIV)/acquired immune deficiency syndrome (AIDS) via theatre [9] and has succeeded in creating social transformation in older adults with vision loss in Canada [10].

Public involvement in designing health communication materials is recognized as central to ensuring that messages are trusted, comprehensible and contextually relevant [11]. Radio and video have been successfully used in Africa for eye health education [12, 13] and community co-creation as well as participatory evaluation have shown to be beneficial in tailoring the intervention to the local sociocultural context in the Zanzibar Arts for Children’s Eyesight (ZANZI-ACE) programme where locally composed songs conveyed eye health messages in Zanzibar [14]. However, in many low- and middle-income countries (LMICs), the structures for meaningful involvement, especially in creative health education, remain underdeveloped. Inclusive involvement of citizens and artists in the judging and validation of health messages offers a promising but underexplored approach to sharing power in communication design. Participatory approaches in health communication have traditionally focused on co-design during early stages of intervention development, and far less attention has been given to how communities can influence the selection, endorsement and refinement of final communication outputs. In most settings, these decisions remain expert-led. Rather than limiting public input to early development stages, inclusive participatory judging enables communities to influence which messages are retained, revised or prioritized for dissemination. This represents a shift from consultation to shared authority in health communication design.

**Fig. 1:**
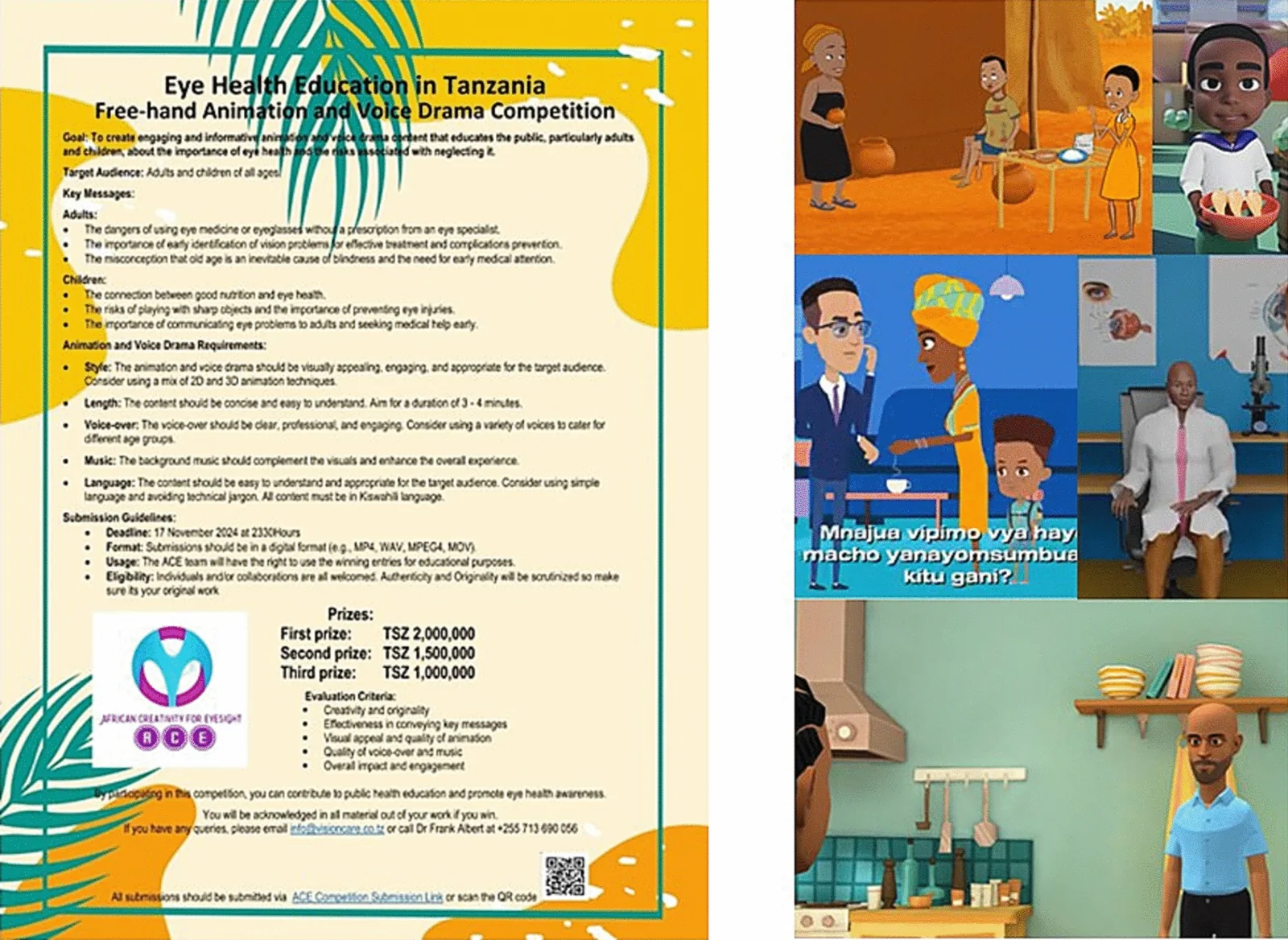
Screenshots of second- and third-place animations. Still frames illustrating key scenes from the two highest-rated animation submissions (Artwork 3 and Artwork 1), depicting culturally relevant settings and visual storytelling approaches used to convey eye-health messages

This study forms part of the African Creativity for Eyesight (ACE) programme, a participatory initiative that uses animation, and voice drama to promote eye health in Dodoma, Tanzania. The ACE project was divided into three phases: phase 1 involved identification of barriers to service uptake and message development through community stakeholder workshops. Supplemental File 1 features a table containing the messages co-developed with the participants in this phase. Phase 2 focused on production of the educational artworks by local creatives through a nationwide competition and the selection of the best entries by a judging panel. Figure 1 shows the competition advertisement and screenshots from the animation entries. Phase 3 is a validation phase focusing on how well the eye health messages in the three winning artworks were delivered.

This paper examines two key questions:

How did inclusive participatory judging and validation support public involvement and co-production in the development of eye health messages?

What were participants’ experiences of inclusion, confidence and influence within these processes?

Operational mechanisms for embedding inclusive public involvement into routine health communication decision-making are generally limited. By focusing on how communities engaged as co-judges and decision-makers, this paper contributes new evidence on participatory and inclusive approaches to health communication in LMICs, highlighting how inclusive shared evaluation can foster ownership, trust and contextual relevance in public health messaging.

## Methods

### Study design

This study adopted a PAR design, embedded within the African Creativity for Eyesight (ACE) programme in Tanzania. The ACE initiative aims to promote community eye health through creative media including animation, music and voice drama. This study focused on two participatory phases, the judging and validation, which actively involved community members, artists and public representatives in evaluating and refining health messages created through a national arts competition (Fig. 1). The research team used a mixed-methods approach combining quantitative scoring of messages with qualitative reflections from participants. Quantitative scoring was used to facilitate group deliberation and not to measure effect size or make population-level estimations. Descriptive statistics were therefore used to summarize collective judgements and prioritization outcomes. Inferential statistical testing was not conducted, as the data were derived from purposively sampled participants, reflected non-independent deliberative processes and were not intended to support hypothesis testing.

### Public and patient contribution

The study was co-designed with the Ministry of Health and local stakeholders, who identified the need for culturally grounded and accessible messages about childhood eye health. Public involvement took place at multiple levels:

- Creative production: Local artists and storytellers interpreted key eye health themes into animated and voice drama scripts.
- Judging: A panel of 14 community judges, comprising teachers, parents, artists, youth representatives and people with disabilities, scored each submission based on predefined criteria including accuracy, clarity, cultural relevance and inclusivity.
- Validation: A subsequent workshop with 17 participants, including community members and public health officers, reviewed the top three entries for message inclusion, comprehension and effectiveness.

Public participants were co-decision-makers whose input shaped which messages were refined and approved for dissemination. They also provided narrative reflections on accessibility and representation, particularly for people with hearing or visual impairments.

### Setting

The study was conducted in Dodoma, which is home to government facilities, universities, schools for the blind, and a varied population consisting of many cultures and religions, rural and urban communities and numerous artists. The city was chosen for its favourable environment for cross-sector collaboration to create opportunities for improved sustainability, knowledge exchange and stronger community ties [15].

### Sampling and recruitment

A total of 14 community judges (7 women and 7 men) and 17 validation workshop participants (9 women and 8 men) took part in the study. There was one deaf and one blind participant in both the judging and validation phases. Participants were purposively selected to ensure diversity in gender; age; lived experience, including that of persons with disabilities; and professional background relevant to health communication or community engagement. (Table 1). Participants were identified through the Ministry of Health in collaboration with regional and district health and education officers, who drew on existing community and professional networks. These included representatives from organizations of persons with disabilities (OPDs), schools, local artists, parents, youth groups and health service providers. Inclusion criteria were (1) adults representatives aged 18 years and above, (2) child and youth representatives included for perspective, (3) ability to participate in group discussions in Kiswahili and (4) willingness to engage in both scoring and discussion processes. No formal exclusion criteria were applied beyond availability and ability to provide informed consent; however, efforts were made to avoid over-representation from any single stakeholder group. Written informed consent was obtained from all participants after explaining study aims and confidentiality procedures in Swahili.

**Table 1:** Participant profile and justification for inclusion in judging and validation workshops

Participant role	Number of participants						Justification as a panel member
	Judging			Validation			
	Male	Female	Total	Male	Female	Total	
University lecturer/researcher	0	1	1	1	1	2	To evaluate the quality of the graphics and audio content in the submissions
Animation/voice drama freelancer	1	0	1	0	0	0	To evaluate the quality of the graphics and audio content in the submissions
Ministry of health	1	1	2	1	2	3	To evaluate the quality and accuracy of the eye health messages
Organizations of persons with disabilities	1	0	1	1	0	1	To evaluate the accessibility and appropriateness of the content for the target audience
Eye health NGO	2	2	4	2	2	4	To evaluate the quality and accuracy of the eye health messages as well as the accessibility and appropriateness of the content for the target audiences
Eye programme co-ordinator	0	1	1	0	1	1	To evaluate the quality and accuracy of the eye health messages
Parents	1	1	2	1	1	2	To evaluate the comprehension and effectiveness of messages for adults and children
Students, some with vision and hearing impairment	1	1	2	1	1	2	To evaluate the comprehension and effectiveness of messages for children and accessibility for people with vision impairment and hearing loss
Youth out of school, some with vision and hearing impairment	0	0	0	1	1	2	To evaluate the comprehension and effectiveness of the messages as well as accessibility for people with limited formal education
Total	7	7	14	8	9	17	

NGO, non-governmental organization

### Judging process

The nationwide free-hand animation and voice drama competition gave artists 2 months to submit 3–4-min Kiswahili artwork pieces, with a top prize of TZS 2,000,000 (US$ 763). A full day judging event conducted in Kiswahili took place on 19 February 2025. Each entry was reviewed in a separate session, starting with an anonymous introduction to the artwork to prevent bias. Facilitators explained the judging process before revealing the artwork ID for each piece and playing the content. Participants scored each piece, viewed it a second time to revise scores if needed and, lastly, had a group discussion before moving on to the next submission. Students with vision or hearing impairment were helped by a dedicated and experienced vision or deaf support assistant from their school. The judging questionnaire was distributed in paper format and captured the artwork’s ID, title and date. The judges used 10 evaluation criteria on a scale of 1–10 (1–3 = poor, 4–6 = average, 7–9 = good and 10 = excellent) and added comments for each. Supplemental File 2 shows the English version of the template judges used to assess the artworks.

We calculated the sum of the total score out of 100 from each judge, and the three highest scoring entries were deemed winners and proceeded to the validation phase. A mean score was calculated for each criterion in all artworks. Comments were translated from Swahili to English using online software and verified by a native Swahili speaker from the local team. Common ideas expressed in the comments were summarized and a frequency of the ideas was calculated to develop structured feedback for the artists.

### Validation process

The validation took place in Kiswahili during a half-day event. Each session began with an introduction to the winning entries and instructions from the facilitators. Participants watched and listened to the artwork, completed the evaluation and then reviewed the piece again with the option to revise their responses. The presence of dedicated and experienced vision and deaf support assistants enabled participants with hearing or visual impairment to contribute. Each validation took 45–60 min to complete, ending with a group discussion. The validation form was distributed in paper format. It contained five sections: (A) respondent details and title of the material; (B) instructions; (C) closed questions on message inclusion, comprehension and effectiveness of delivery, using “yes”/“no”; (D) closed questions on message inclusion, comprehension and effectiveness of delivery using 3-point Likert scales for adult and child messages; and (E) open-ended questions on message clarity, audience appropriateness, accessibility and suggestions for improvements. Supplemental File 3 shows the English version of the validation questionnaire where participants scored the artworks and wrote their comments.

### Data analysis

Responses were entered into a Microsoft Excel spreadsheet. The inclusion, comprehension and effectiveness of each message in the three winning artworks were analysed. For inclusion and comprehension, percent agreement was calculated using the number of participants who selected “yes” divided by the total number of responses. For message effectiveness, the scores of “very effective” = 2, “somewhat effective” = 1, and “not effective” = 0 were used, and percent agreement was calculated using the total score divided by the maximum possible score. A mean inclusion, comprehension and effectiveness score was calculated for each artwork. Responses not rated or marked as “NA” were not included. The results of the co-evaluation processes were shared with artists to aid revision of the artworks based on participant feedback. Qualitative data, field notes, participant reflections and group discussions were transcribed verbatim, translated into English and thematically analysed using Braun and Clarke’s framework [16]. Themes were developed inductively around three domains: (1) message clarity and resonance, (2) inclusivity and accessibility and (3) experience of involvement and influence. Common ideas expressed in the open-ended questions were summarized, and a frequency was calculated to analyse the reasons behind panel ratings.

### Participant feedback

An essential component of PAR is the perception of the participants that their time and contributions are truly valued. To evaluate the collaborative environment of the judging workshop and assess participant engagement, feedback was collected using a nine-question form based on a 5-point Likert scale ranging from “strongly agree” to “strongly disagree”. The proportion of responses in each Likert category was then calculated to analyse response distribution.

### Researcher reflexivity

The facilitation team brought diverse professional and cultural perspectives to the study. The lead facilitator was a Global Eye Health researcher based in the UK, with 18 years of experience in community eye health research and a decade of fieldwork in sub-Saharan Africa. He was joined by a public ophthalmologist from the University of Dodoma and a public health optometrist with over 20 years of experience in eye care service delivery and health promotion in Tanzania. Together, they co-facilitated the judging and validation sessions in Swahili, ensuring local ownership and cultural sensitivity.

Two additional team members, a public health optometrist and a public health specialist, served as note-takers, focusing on participant interactions and contextual observations. A third-year medical student contributed to data management and preliminary analysis under supervision, providing a reflective lens from an early-career researcher’s standpoint.

Throughout the process, the facilitation team maintained reflexive field journals to document observations on group dynamics, power relations and how their professional backgrounds might shape interpretation. Regular daily debriefs were held to discuss these reflections and ensure that participants’ voices, rather than researchers’ preconceptions, guided the analysis and interpretation.

The team anticipated that power dynamics may emerge between UK-based and Tanzanian researchers throughout the research process. Efforts were made to equalize these by having a senior author from both countries, ensuring equal access to the data collected for the entire team, which required navigating language barriers as well as meaningful two-way exchange of knowledge and skills with the Tanzanian team contributing linguistic, cultural and research expertise.

The facilitators were aware that the young people with disabilities may face additional barriers to participating in the judging and validation processes and liaised closely with the communication support workers to ensure everyone had equal chance to contribute.

## Results

The panels represented a wide range of perspectives including those of researchers, artists, Ministry of Health staff, members of OPDs, non-governmental organizations (NGOs), parents, students and out-of-school youth (Table 1). This diversity was intentionally sought to ensure that multiple community voices shaped the judging process and interpretation of messages. The competition received 11 entries: 9 animations and 2 voice dramas. (Fig. 1) Of the 11 artworks, 4 targeted adults only, 1 targeted exclusively children and 6 targeted both adults and children.

## Judging results

The three highest-scoring artworks were Artwork 5 (voice drama, adults and children), Artwork 3 (animation, adults) and Artwork 1 (animation, adults and children), with total scores of 87.22, 83.19 and 77.85, respectively (Table 2). Beyond numerical ranking, the judging discussions revealed a shared evaluative logic in which accessibility, cultural realism and ethical reach consistently outweighed technical polish or aesthetic appeal. While many pieces were praised for being engaging and culturally relevant, recurring concerns included limited accessibility – particularly for people with sensory disabilities, including people with vision and hearing impairment. Judges noted, for instance, that “it is not accessible for deaf people; video needed” and “lacks sign language.”

**Table 2:** Judging scores and qualitative feedback for 11 submitted artworks

Artwork ID	Type of artwork	Target	Mean score (maximum = 10)											Qualitative feedback (*n *= frequency of mentions)
			1^*^	2	3	4	5	6	7	8	9	10	Total	
1	Animation	Adults and children	7.86	8.00	8.50	7.29	8.43	6.92	7.57	8.21	7.43	7.64	77.85	Eye problems, treatment and treatment outcomes unclear (*n *= 5) Limited suitability for children (*n *= 3) Rural setting only (*n *= 3) Music not appropriate for all regions of Tanzania (*n *= 2) Easy to understand (*n *= 1) Inaccessible to people with low vision (*n *= 1)
2	Voice drama	Adults and children	7.92	6.64	8.21	7.00	7.50	7.14	7.21	7.00	7.07	7.14	72.83	Incomplete (*n *= 4) Fits cultural context (*n *= 2) Inaccessible to people with hearing impairment (*n *= 2) Opportunity for social media distribution (*n *= 1) Easy to understand (*n *= 1) Radio is outdated (*n *= 1) Not culturally relevant (*n *= 1)
3	Animation	Adults	8.85	8.14	8.07	7.93	8.64	8.14	7.93	8.21	8.85	8.43	83.19	Improve visuals (*n *= 4) Clarify messages (*n *= 3) Importance of nutrition shown (*n *= 2) Sustainable (*n *= 2) Includes several messages (*n *= 2) Improve accessibility for deaf people (*n *= 2) Improve delivery for children (*n *= 2) Inclusive (*n *= 1) Educates well on prevention (*n *= 1)
4	Animation	Children	6.86	6.93	6.64	5.86	6.14	5.43	5.79	5.86	5.86	5.71	61.08	Not culturally appropriate (*n *= 7) Inaccessible, unappealing and unclear imagery (*n *= 5) Incorrect message (*n *= 3) Lacks sign language (*n *= 1)
5	Voice drama	Adults and children	8.71	8.86	9.21	8.64	8.93	8.36	9.00	8.29	8.29	8.93	87.22	Not accessible for deaf, video needed (*n *= 5) Several clear messages included (*n *= 2) All audiences (*n *= 2) Refine dialogue (*n *= 1) Culturally appropriate (*n *= 1)
6	Animation	Adults	8.14	6.29	7.36	5.64	5.79	6.36	7.00	6.93	5.86	6.21	65.58	Several messages included (*n *= 3) Not appealing (*n *= 3) Inappropriate attire (*n *= 3) Inaccessible for hearing impaired (*n *= 2) Professional appearance (*n *= 1) Easy to understand (*n *= 1)
7	Animation	Adults and children	7.57	6.57	7.00	6.57	7.07	6.21	6.71	6.79	7.43	6.93	68.85	Clear messages (*n *= 3) Inappropriate language (*n *= 2) Improve graphics (*n *= 2) Good animation style (*n *= 1) No sign language (*n *= 1) Advice outside intended purpose (*n *= 1)
8	Animation	Adults	5.36	4.57	4.43	3.57	4.29	4.07	4.14	4.29	3.57	4.29	42.58	Lacks sound (*n *= 4) Incorrect spelling/grammar (*n *= 3) Lacks cultural relevance (*n *= 1)
9	Animation	Adults and children	7.36	5.79	7.29	6.21	6.57	6.43	6.21	6.50	6.29	7.00	65.65	Appealing images (*n *= 2) Messages need improvement (*n *= 2) Inappropriate language (*n *= 1)
10	Animation	Adults and children	8.36	6.50	7.57	7.07	7.29	6.79	6.86	7.07	7.14	6.71	71.36	Encourages good nutrition (*n *= 3) Effective (*n *= 2) Harmful practices not shown (*n *= 2) Encourage food over supplements (*n *= 1) Urban setting only (*n *= 1) Child appears too old (*n *= 1) Poor spelling (*n *= 1)
11	Animation	Adults	7.00	6.14	6.14	6.21	6.43	5.57	5.64	6.07	6.50	6.29	61.99	Discourages use of traditional medicine (*n *= 3) Visuals require improvements (*n *= 2) Promotes seeking hospital eye care (*n *= 1) Culturally appropriate (*n *= 1) Incomplete (*n *= 1) Patriarchal story (*n *= 1)
All submissions			7.64	6.77	7.31	6.54	7.01	6.49	6.73	6.84	6.75	6.84	68.92	

Discussions during and after judging revealed a shift from simply identifying flaws to exploring collective solutions. Participants repeatedly emphasized accessibility as a shared ethical responsibility, not a technical afterthought. One female musician explained: “We understood that we cannot reach everyone using a single format. But they must show how people see themselves and their realities in the animation or voice drama.”

Participants also recognized that combining formats, voice and visual could expand reach across literacy and ability groups, and an evolving awareness of inclusivity and co-ownership in the creation of health messages was further demonstrated when concerns around cultural appropriateness and production quality were voiced. Judges highlighted elements such as “inappropriate attire”, “music not suitable for all regions” and *“poor spelling”* as detracting from authenticity and credibility. Rather than being framed as criticism, these reflections became part of a collaborative dialogue between community members, artists and facilitators, allowing for culturally grounded refinement of the materials.

## Validation results

In the validation phase, the three shortlisted entries were reassessed for message inclusion, comprehension and effectiveness. Voice Drama 5 performed strongest across all domains (97.1%, 94.8%, 87.7%), followed by Animation 3 (89.6%, 90.9%, 75.4%) and Animation 1 (44.9%, 60.0%, 45.6%) (Table 3). Participants interpreted these differences not as indicators of intrinsic “quality”, but as reflections of how closely each format aligned with everyday communication practices, sensory accessibility and recognizable social contexts. Participants praised the top voice drama for its relatability and simplicity and felt a familiarity with the format due to a strong culture of oral storytelling in Tanzania. They again called attention to the exclusion of deaf audiences and recommended adding video, sign language and clearer dialogue. Similarly, Animation 3 was valued for its concise messaging and visual appeal, yet participants proposed refinements to ensure visual realism (“match speech and visuals”) and cultural sensitivity (“avoid Westernised settings”). Animation 1 generated the most discussion. Participants commented on its confusing storyline, outdated visuals and inaccessible design, recommending “subtitles, sign language, and a clearer story showing everyday life.”

**Table 3:** Validation questionnaire responses for the top three artworks

Artwork ID and type	Message	Inclusion score (%)	Comprehension score (%)	Effectiveness score (%)	Appropriateness for target audience^*^	Accessibility^*^	Qualitative feedback^*^
1 – Animation	1	87.5	87.5	66.7	Yes (*n *= 8) No (*n *= 4) For children only (*n *= 5) For children and adults (*n *= 3)	Inaccessible to the deaf (*n *= 5) Inaccessible to people who cannot read (*n *= 2)	Add subtitles, sign language and voice-over (*n *= 16) Messages lack clarity (*n *= 14) Improve graphics (*n *= 8) Include all messages (*n *= 5) Unrealistic setting (*n *= 3) Make more concise and add a conclusion (*n *= 3) Emphasize early identification and attendance of follow-up appointments (*n *= 3)
	2	60.0	69.2	57.1			
	3	14.3	33.3	19.2			
	4	7.1	33.3	23.1			
	5	7.1	36.4	30.8			
	6	93.6	100	76.7			
	All message mean	44.9	60.0	45.6			
3 – Animation	1	100.0	93.8	86.7	Yes (*n *= 9) No (*n *= 0) For adults and children (*n *= 1)	Yes (*n *= 1) Inaccessible to the deaf (*n *= 2) Clear and simple language used (*n *= 2)	Inappropriate clothing and setting (*n *= 12) Clarify message on balanced diet and dangers of sharp objects (*n *= 10) Health care provider should encourage the whole community to go for regular eye exams at an eye clinic (*n *= 8) Add subtitles, sign language and voice-over (*n *= 8) Match speech and visuals (*n *= 5) Do not mix English and Swahili (*n *= 2) Includes several clear messages (*n *= 2)
	2	81.3	87.5	73.3			
	3	75.0	84.6	59.4			
	4	100.0	100.0	80.0			
	5	87.5	85.7	71.4			
	6	93.8	93.8	81.3			
	All message mean	89.6	90.9	75.4			
5 – voice drama	1	88.2	88.2	82.4	Yes (*n *= 12) No (*n *= 0) For adults and children (*n *= 5)	Inaccessible to the deaf (*n *= 2)	Unrealistic scenarios (*n *= 11) Include more messages (*n *= 7) Difficult to understand (*n *= 7) Add video (*n *= 4) Add sign language and subtitles (*n *= 3) Shows real-life situations (*n *= 2) Add a summary of guidelines (*n *= 2) Lacks a conclusion (*n *= 1)
	2	100.0	100.0	85.3			
	3	94.1	100.0	93.8			
	4	100.0	94.1	97.1			
	5	100.0	100.0	79.4			
	6	100.0	86.7	88.2			
	All message mean	97.1	94.8	87.7			

Across the validation discussions, participants described a shift from initially assessing messages as “good” or “bad” towards collectively interrogating *who* each message reached, *who* it excluded and *why*. This progression reflected a learning process in which evaluation became increasingly relational and systems-aware rather than purely content-focused. Feedback converged around three participatory insights:

- Accessibility and inclusion: Materials should reflect the needs of people with sensory and literacy differences.
- Cultural ownership: Messages are most powerful when communities can “see themselves” in the stories.
- Collaborative learning: Reviewing the materials together deepened understanding of eye health and strengthened participants’ sense of contribution.

## Judging workshop experience feedback

Quantitative feedback underscored the sense of inclusion and agency. All participants agreed that the topics were important and that the sessions enhanced their understanding of children’s eye health. The high levels of agreement across Likert-scale responses (Table 4), particularly regarding confidence (92.9%) and feeling valued (100%), were reflected in accounts describing a strong sense of ownership and agency. Participants frequently attributed their confidence to the structured, participatory nature of the process, where scoring and discussion enabled them to see how their input directly influenced collective decisions. In this way, the quantitative ratings did not merely indicate satisfaction but corresponded with deeper qualitative expressions of empowerment, shared authority and inclusion. More importantly, participants framed this confidence as emerging *through* the collective process rather than from prior expertise, repeatedly noting that the structured discussion and shared authority enabled them to contribute on equal footing with professionals. Participants also described the experience as empowering: “It was the first time I realised my opinion can shape what others will learn”, said one student participant. Another youth participant noted, “We felt equal, even though some were doctors and others were just parents.” The blind participant found the process interesting and stated this was the first time she was asked for her input. She added, “At first, it was difficult to give an opinion but with the help of the assistant, I was able to do so.” The deaf child stated, “I enjoyed the animation and I was happy to see another child in the panel.” These reflections illustrate that the participatory structure of the judging process. Its emphasis on discussion, consensus and shared authority helped transform evaluation into a collective learning experience. Minor dissenting views were noted, mainly regarding limited time for discussion, but no participant expressed discomfort or regret for taking part.

**Table 4:** Judging workshop feedback on participant experience

Statement	Strongly agree (%)	Agree (%)	Neutral (%)	Disagree (%)	Strongly disagree (%)	Not rated (%)
The topics were important.	85.7	14.3	0	0	0	0
The information shared increased my understanding.	64.3	28.6	0	0	0	7.1
There was enough time to participate and share my ideas.	85.7	7.1	0	7.1	0	0
My contribution was recognized and valued.	85.7	14.3	0	0	0	0
I felt comfortable.	92.9	7.1	0	0	0	0
I felt confident to participate.	92.9	7.1	0	0	0	0
My knowledge and experience were valued.	85.7	7.1	0	0	0	7.1
It gave me an opportunity to contribute.	85.7	14.3	0	0	0	0
It changed my understanding about eye health for children.	100	0	0	0	0	0

Taken together, the quantitative scores functioned less as performance metrics and more as boundary objects that structured dialogue, comparison and reflection among diverse stakeholders. It was through this iterative combination of scoring, discussion and revision that shared priorities, particularly accessibility and cultural credibility, were surfaced and negotiated.

## Discussion

This study explored how participatory judging and validation of locally produced animations and voice dramas can embed public involvement in developing eye health education materials in Tanzania. The process demonstrated that community-led evaluation is not only feasible in low-resource contexts but also instrumental in improving the inclusivity, cultural authenticity and technical quality of health communication. Had the evaluation been conducted solely by medical professionals, the review might have focused on message accuracy alone, overlooking the production aesthetics, nuanced cultural and emotional aspects that artistic and community members identified as vital to engagement.

While voice drama received the highest scores for message inclusion, comprehension and effectiveness, participants’ discussions revealed why this format resonated most. They described how familiar oral storytelling, sound effects and humour conveyed empathy and realism that mirrored Tanzanian life, echoed by the findings of Meppelink et al. [17] and in sexual health interventions [18, 19]. Farr et al. examined the influence of an HIV/AIDS radio campaign in Ethiopia, finding a significant correlation between liking the campaign and reports of behaviour change, showing the importance of creating materials which appeal to the target audience [20]. If this had been a standard medical review, voice drama might have been ranked highly for simplicity but without uncovering these relational dynamics that make stories meaningful and memorable.

In contrast, animation entries displayed greater variability in clarity, tone and visual realism. Some animations were praised for being colourful, creative and educational, but others were criticized for unrealistic attire, Westernized music or mismatched visuals and speech. These issues, while seemingly technical, became springboards for rich conversations about cultural representation. Participants debated whether characters should wear local clothing, and whether background settings should show markets, farms or urban neighbourhoods. Such reflections illustrate how artistic critique became a form of social dialogue, linking aesthetic choices to cultural belonging. Future competitions should also explicitly encourage nationwide relevance and consider integrating iterative feedback cycles before the validation phase as well as performed by Racicot-Matta et al. [21].

Accessibility emerged as both a design challenge and a shared ethical commitment among both the three participants with hearing and vision impairment and those without disabilities. Judges highlighted recurring gaps for people who are deaf, are blind or have low literacy, calling for subtitles, sign language, comprehensive voice-overs and clearer visual cues. Importantly, they framed accessibility as a collective responsibility rather than a technical add-on. A shared view among participants was that visual appeal alone was insufficient; every viewer, regardless of ability or literacy, needed to be able to understand the message. Without this participatory emphasis, accessibility might have been treated as an afterthought in post-production rather than a guiding principle from the outset. The discussion reframed inclusion as integral to the creative ethos of public health messaging.

The PAR framework underpinned this inclusive environment. Conducting the sessions in Kiswahili and having facilitation led by a local eye health non-governmental organization helped flatten hierarchies between researchers, artists and community representatives. Participants reported feeling respected, valued and confident in contributing to design decisions. Had the judging been managed in English or under external facilitation, non-professional voices, especially those of parents, youth and people with disabilities, might have been marginalized. Instead, the PAR design turned evaluation into co-learning: participants not only assessed materials but collectively explored how visual, linguistic and emotional cues shape public understanding of eye health.

Beyond immediate outputs, the inclusive participatory judging process also improved process literacy among artists and community members alike. Discussions revealed that many artists had limited experience translating biomedical messages into creative form. By interacting directly with judges, they received grounded feedback that bridged artistic imagination with public health accuracy. If this iterative exchange had not occurred, future productions might have repeated the same technical and cultural gaps, perpetuating weak message quality across campaigns. Structured supports, such as feedback templates, local mentorship or pre-submission clinics, could further strengthen this creative–public-health interface.

These findings contribute to broader debates about operationalizing meaningful public involvement in LMICs, where formal patient and public involvement (PPI) structures are still evolving. The judging and validation model offers an inclusive practical, scalable mechanism that embeds power-sharing in decision-making. It shows that communities can credibly evaluate the educational, aesthetic and ethical quality of health media when provided with inclusive facilitation and clear frameworks. Had message validation been left to external experts alone, the outputs would likely have reflected institutional perspectives rather than lived realities, undermining the relevance and uptake of national health campaigns.

## Strengths and limitations

A major strength of this study is its integration of quantitative validation with qualitative insight, enabling an understanding of both the effectiveness of the creative materials and the experiential impact of participation. The inclusion of multiple stakeholder perspectives, artists, health professionals, people with disabilities and community members, brought diverse expertise to defining what makes messages effective, engaging and fair. If the study had relied solely on numerical scores, it would have produced rankings without the contextual learning that gives meaning to those numbers. A limitation, however, was the small number of participants, especially those with disabilities, which constrained the depth of accessibility analysis and generalizability of our study. This could be improved in the future through further engagement with local special schools and parents of children with disabilities. Additionally, the workshops’ short duration and limited long-term follow-up make it difficult to understand how participation influenced subsequent creative practice or advocacy. Our study only included quantitative feedback on participation experience in the judging phase. This should be extended to the validation stage in future studies, include statements about how well participants with disabilities were included and be accompanied by individual qualitative exploration of the panels’ experiences to enable more detailed feedback and avoid social desirability bias.

## Implications for practice

For practitioners and policy-makers, these findings demonstrate that participatory judging can serve as an adaptable model of public involvement in health communication, especially where advisory boards or PPI frameworks are absent. It offers a structured, inclusive and culturally sensitive approach that balances community voice with technical rigour. Ministries and NGOs could integrate judging and validation cycles into media commissioning processes by embedding structured community judging panels at key stages of content development to ensure that public education materials reflect community viewpoints and daily life. For example, district-level workshops could be convened to review draft materials prior to national dissemination, ensuring inclusive, cultural and contextual alignment. NGOs and implementing partners could adopt simplified judging frameworks, such as the 10-criteria scoring tool used in this study, to facilitate community-led evaluation in low-resource settings. Future adaptations should prioritize inclusion of people with disabilities through targeted recruitment and accessible formats (for example, sign language interpretation, audio description) and incorporate iterative feedback loops between community panels and content creators. Without such participatory loops, campaigns risk producing content that informs without inspiring, potentially widening the gap between awareness and action.

## Conclusions

Inclusive, participatory judging and validation transformed community members from recipients of information into co-evaluators of health messaging. Had the project followed a conventional, expert-led pathway, these outputs might have been technically sound yet socially flat – failing to capture the textures of Tanzanian life that make messages believable. By embedding shared reflection and decision-making, this inclusive participatory approach elevated health communication from mere information delivery to an act of collective authorship. Future programmes should continue to balance creative freedom with structured guidance and integrate community stakeholder voices in media evaluation, embedding inclusivity accessibility and cultural authenticity as foundations rather than aspirations. In doing so, participatory arts-based methods can help shift global health education from inclusion by invitation to inclusion by design.

## Supplementary Information

Supplementary Material 1.

Supplementary Material 2.

Supplementary Material 3.

## Data Availability

All data supporting the findings of this study are presented within the manuscript.
