# Supplementary material for: Co-evaluating health messages through inclusive participatory judging and validation: A public involvement study in Tanzania’s eye health education programme
Source: Health Res Policy Syst. 2026 Aug 7;24:95. doi: 10.1186/s12961-026-01519-9 (PMC13737507; doi:10.1186/s12961-026-01519-9)
Supplement: Supplementary file 1 [file 12961_2026_1519_MOESM1_ESM.docx]

| **Target Audience** | **Message** |
| --- | --- |
| Adults | Using eye medicine/eyeglasses without a prescription from an eye specialist is dangerous. |
|  | Early identification of vision problems is an important step in treatment. |
|  | Old age is not a reason for blindness; take the parent/guardian to the hospital early for proper treatment. |
| Children | Get good nutrition to maintain your eye health. |
|  | Avoid playing with sharp objects to prevent eye injuries. |
|  | Speak up about your eye problems! And go to the hospital for treatment. |

Supplemental File 1. Eye health messages
