# Supplementary material for: Co-evaluating health messages through inclusive participatory judging and validation: A public involvement study in Tanzania’s eye health education programme
Source: Health Res Policy Syst. 2026 Aug 7;24:95. doi: 10.1186/s12961-026-01519-9 (PMC13737507; doi:10.1186/s12961-026-01519-9)
Supplement: Supplementary file 2 [file 12961_2026_1519_MOESM2_ESM.docx]

| **Criteria** | **Description** |
| --- | --- |
| **1. Relevance to Eye Health** | The material effectively conveys key eye health messages and aligns with the target audience (children/adults). |
| **2. Creativity and Originality** | The content demonstrates creativity, originality, and innovation in the approach to delivering eye health messages. |
| **3. Clarity of Message** | The message is clear, easy to understand, and culturally appropriate for the audience in Tanzania. |
| **4. Engagement** | The material is engaging, captivating, and maintains the audience’s attention. |
| **5. Cultural Sensitivity** | The material respects Tanzanian cultural norms and values while addressing potential socio-cultural barriers to eye health. |
| **6. Quality of Production** | The technical quality of the animation/voice drama, including sound, visuals, and overall presentation. |
| **7. Scalability and Applicability** | The material can be adapted or used for a broader audience or in similar contexts to spread eye health awareness. |
| **8. Impact Potential** | The potential of the material to inspire action, such as improving knowledge, attitudes, or behaviour towards eye health. |
| **9. Inclusivity and Accessibility** | The material is inclusive and accessible to individuals of varying literacy levels, abilities, and diverse backgrounds, ensuring wide reach. |
| **10. Sustainability** | The potential for the material to have a long-term impact and remain relevant for eye health education in the future. |

Supplemental File 2. Judging questionnaire criteria
