# Supplementary material for: Co-evaluating health messages through inclusive participatory judging and validation: A public involvement study in Tanzania’s eye health education programme
Source: Health Res Policy Syst. 2026 Aug 7;24:95. doi: 10.1186/s12961-026-01519-9 (PMC13737507; doi:10.1186/s12961-026-01519-9)
Supplement: Supplementary file 3 [file 12961_2026_1519_MOESM3_ESM.docx]

## Supplementary File 3. Message Validation Questionnaire
